# Research on Image Encryption with Multi-Level Keys Based on a Six-Dimensional Memristive Chaotic System

**DOI:** 10.3390/e27111152

**Published:** 2025-11-13

**Authors:** Xiaobin Zhang, Yaxuan Chai, Shitao Xiang, Shaozhen Li

**Affiliations:** School of Electrical and Electronic Engineering, Wuhan Polytechnic University, Wuhan 430048, China; hb_whpu_xb@163.com (X.Z.); yaxuan_chai@163.com (Y.C.); 22113009@whpu.edu.cn (S.X.)

**Keywords:** chaotic index scrambling, key, six-dimensional memristive chaotic system, Zigzag scrambling

## Abstract

To address the security of digital images, this paper proposes a novel image encryption algorithm based on a six-dimensional memristive chaotic system. First, the algorithm uses the Secure Hash Algorithm 256 (SHA-256) to generate a hash value, from which the initial dynamic key is derived. Next, it integrates Zigzag scrambling, chaotic index scrambling, and diffusion operations to form an encryption scheme with multiple rounds of scrambling and diffusion. In this framework, after each encryption operation, a part of the dynamic key is changed according to the input parameters, and the six-dimensional memristive chaotic system continues iterating to generate the pseudo-random sequence for the next operation. Finally, the proposed algorithm is evaluated using indicators including information entropy, histograms, the Number of Pixels Change Rate (NPCR) and Unified Average Changing Intensity (UACI), encryption time, and so on. The results show that the information entropy of the encrypted image reaches 7.9979; its Chi-square statistic is 186.6875; the average NPCR and UACI are 99.6111% and 33.4643%, respectively; and the encryption time is 0.342 s for the 256 × 256 Cameraman image. These indicate that image encryption is not only effective in encrypting images but also resistant to many conventional attacks.

## 1. Introduction

With the rapid development of Internet communication technologies, the volume of multimedia files such as images and videos which carry critical network information has dramatically increased. Digital images have emerged as the most widely used medium for information transmission due to their advantages, including large information capacity, low storage requirements, and easy transmission. Many of these images may contain privacy information belonging to individuals or teams, and their frequent transmission may expose users to privacy breach risks. Therefore, digital image security has attracted considerable attention [1,2,3,4].

To protect the information security of digital images, researchers have been actively exploring various secure and reliable image encryption algorithms. The “scrambling-diffusion” mechanism derived from Shannon’s theory is a typical structure for image encryption [5]. The combination of scrambling and diffusion can not only alter pixel positions but also convert changes in a single pixel into global or local modifications of the image, thereby altering the image information [6,7]. However, encryption schemes solely relying on the basic scrambling-diffusion combination exhibit low security performance. Therefore, chaotic systems are widely applied in digital image encryption due to their characteristics such as extreme sensitivity to initial conditions and system parameters, unpredictable trajectories, and strong pseudorandom output sequences [8].

Researchers investigate the methods for generating chaotic sequences using secret keys and chaotic systems, and thereby achieve image encryption [9,10]. For example, Fridrich [11] firstly designed an encryption scheme based on a two-dimensional chaotic system, addressing the issue of insufficient encryption security at the plaintext and key levels after the discretization of chaotic maps. Gao X [12] designed a novel image encryption algorithm by combining two one-dimensional chaotic maps, resolving the flaws in encryption schemes of chaotic cryptosystems caused by the adoption of one-dimensional chaotic maps lacking complex dynamic behaviors. However, the studies show that low-dimensional chaotic systems (e.g., those used in Refs. [11,12]) have simple structures and small key spaces, making them vulnerable to brute-force attacks and thus difficult to meet the requirements of high security encryption [13,14]. In addition, the security of chaotic image encryption depends on the chaotic system; if the employed chaotic system exhibits poor performance, the image encryption effect will be unsatisfactory.

To enhance the performance of chaotic systems, numerous researchers have begun to design and investigate high-dimensional chaotic systems using the multidimensional extension method and spatial expansion method [15,16]. Among various approaches, memristors exhibit prominent nonlinearity, low power consumption, and scalability, making them highly suitable for constructing high-dimensional hyperchaotic systems with more complex dynamics [17]. For example, Itoh et al. [18] designed several nonlinear oscillator models by combining a memristor model and Chua’s chaotic system, and pioneeringly established memristor-based chaotic systems. Chen M et al. [19] designed a four-dimensional memristive chaotic system by combining a memristor model and a chaotic system, addressing the issue of reduced application security caused by the existence of equilibrium points in the system. Wang Y F et al. [20] integrated two cubic nonlinear flux-controlled memristor models and a four-dimensional chaotic system, designing a novel six-dimensional memristive chaotic system and presenting a synchronization control method. This work resolved the synchronization control challenge of high-dimensional memristive chaotic systems. Then, an increasing number of scholars have proposed digital image encryption algorithms based on high-dimensional memristive chaos due to their excellent performance. For instance, Sha et al. [21] designed a novel image encryption scheme by combining a four-dimensional memristive chaotic system and an alignment-confusion-permutation structure, resolving the issue of insufficient encryption strength in simple encryption systems. In addition, Yang D et al. [22] designed a novel image encryption scheme by combining a four-dimensional memristive chaotic system and compressed sensing technology, addressing the problem of poor image reconstruction quality. Nonetheless, it is worth noting that high-dimensional chaotic systems are highly complex and generate chaotic sequences with higher randomness, which reduces encryption efficiency when they are applied in the field of image encryption (e.g., Refs. [21,22]). Additionally, researchers usually use pseudorandom sequences generated from a single set of initial values in different operations of the encryption scheme (e.g., Ref. [21]). This means that once attackers obtain the initial parameters, they can acquire the sequences used for encryption in each operation, thereby increasing the probability of image decryption.

From above, the work of this paper is paid more attention the following three aspects. First, we utilize a six-dimensional memristive chaotic system with more complex dynamic behaviors and a larger key space to enhance the security of the algorithm. Second, we effectively integrate the hierarchical key stream mechanism with Zigzag transform and improved chaotic index scrambling, and thus design an image encryption algorithm with a scrambling-diffusion process; this mechanism ensures that the sequences used in each step are generated by the chaotic system based on different initial values. Meanwhile, we simplify the encryption scheme to improve its encryption efficiency on the premise of ensuring the security of the algorithm. Finally, we evaluate the security and efficiency of the image encryption algorithm using a series of metrics.

## 2. Basic Theory

### 2.1. Six-Dimensional Memristive Chaotic System

The mathematical model of the six-dimensional memristive chaotic system [20] is presented as follows:

(1) $$\left\{\begin{array}{c} \dot{x}=a\left(y-x\right)\\ \dot{y}=bxW\left(v\right)+8xz\\ \dot{z}=2x^{2}-5xy\\ \dot{w}=cW\left(u\right)y\\ \dot{v}=dx\\ \dot{u}=y \end{array}\right.$$

where the expressions of the two flux-controlled memristor models are $W(v)=m+nv^{2}$ and $W(u)=p+qu^{2}$, respectively, *a*, *b*, *c*, *d*, *m*, *n*, *p*, *q* are system parameters, and x, y, z, w, v, *u* are system state variables; $\dot{x}$,$\;\dot{y}$,$\;\dot{z}$,$\;\dot{w}$,$\;\dot{v}$, and $\dot{u}$ denote the derivatives of the system state variables with respect to time *t*. When the system parameters are set as *a* = 6, *b* = 2, *c* = 0.1, *d* = 2, *m* = −1, *n* = −0.2, *p* = 1 and *q* = 0.1, and the system initial values are given by Y = [0.1, 0.1, 0.1, 1, 1, 1], the Lyapunov exponents for the system are calculated as follows: ${LE}_{1}=0.1421$, ${LE}_{2}=0.0019$, ${LE}_{3}=0.0004$, ${LE}_{4}=-0.0216$, ${LE}_{5}=-0.0994$, and ${LE}_{6}=-6.0234$. According to the Lyapunov dimension calculation equation:

(2) $$D_{L}=L+\frac{1}{\left|{LE}_{L+1}\right|}\sum_{i=1}^{L}{LE}_{i}$$

**Figure 1:**
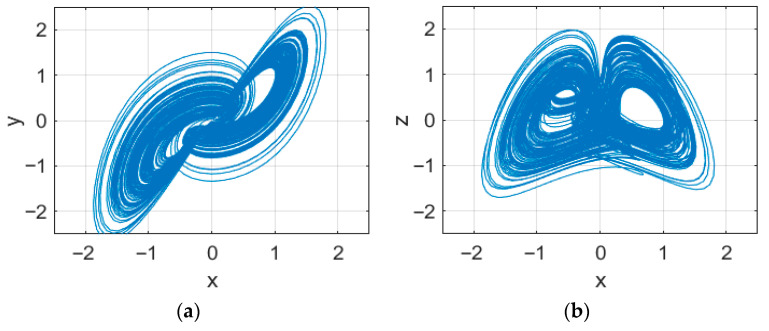

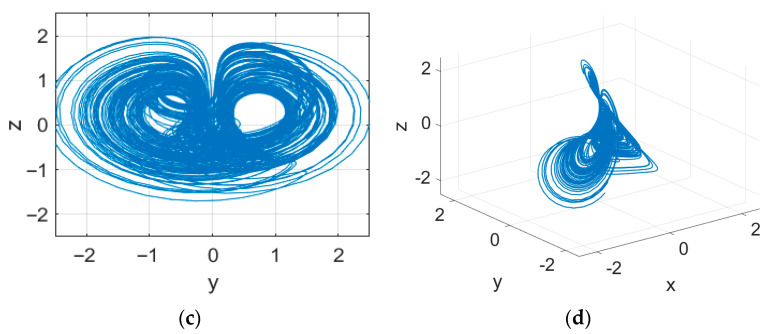
Phase orbits of the chaotic system. (**a**) x-y; (**b**) x-z; (**c**) y-z; (**d**) x-y-z.

In Equation (2), it is *L* = 5, $D_{L}$ = 5.0039. The dimension is fractional, and there are three positive and three negative Lyapunov exponents, thus indicating that this six-dimensional memristive chaotic system is a hyperchaotic system. The phase trajectory diagram, shown in Figure 1, reveals that its dynamical behavior is highly complex and unpredictable, and thus fulfills the requirements of an encryption algorithm.

### 2.2. Zigzag Scrambling

**Figure 2:**
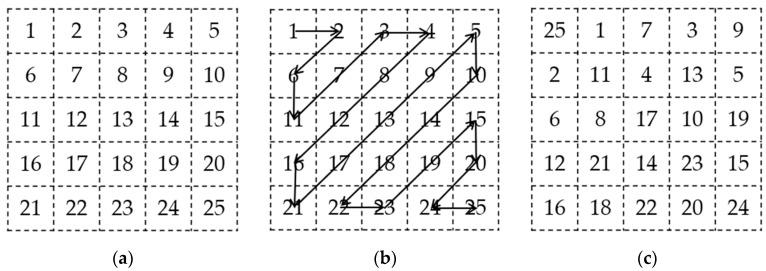
Zigzag scrambling. (**a**) Plaintext Matrix; (**b**) Zigzag path; (**c**) Transformed matrix.

The Zigzag algorithm starts from the first position (1, 1) of the matrix in Figure 2a and traverses the element values in the matrix sequentially along the unique Zigzag path, as shown in Figure 2b. The scanned values are arranged into a one-dimensional array and then recombined into a matrix, as shown in Figure 2c. The Zigzag scrambling algorithm can rapidly and effectively change the original image pixel positions, thus being widely applied in digital image encryption [23]. We employ the Zigzag scrambling algorithm for initial scrambling of the image. Additionally, considering that a single Zigzag scrambling operation yields suboptimal results for image boundaries during processing, we fix the number of Zigzag scrambling iterations at 8.

### 2.3. Chaotic Index Scrambling

The algorithm sorts the chaotic sequence in ascending order to generate a new vector and returns its index numbers. Next, it converts the image matrix into a one-dimensional vector using row-major ordering, performs pixel scrambling based on the index numbers to produce a new one-dimensional vector, and finally reconstructs this new vector back into an image matrix.

**Figure 3:**
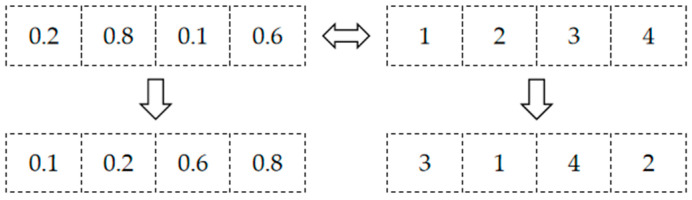
Schematic diagram of indices generation.

As shown in Figure 3, the algorithm sorts the vector [0.2, 0.8, 0.1, 0.6] in ascending order to yield [0.1, 0.2, 0.6, 0.8], from which indices are derived. These indices record the original position of each element within the new vector. Subsequently, all elements of the one-dimensional vector derived from the image matrix are rearranged via the index-based mapping, ultimately generating the scrambled image. In this study, we utilize sequences *v* and *u* to execute this scrambling process.

## 3. Encryption Algorithm

### 3.1. Image Encryption Algorithm Design

**Figure 4:**
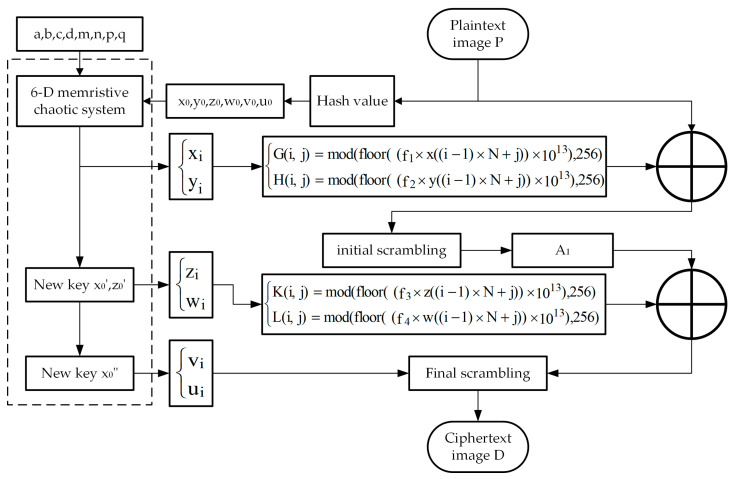
Flowchart of the encryption algorithm.

Step 1: Read the plaintext image P(M×N), with *M* and *N* denoting the length and width of the image, respectively (Figure 4).

Step 2: Take the input key of the algorithm as system parameters *a*, *b*, *c*, *d*, *m*, *n*, *p*, and *q*, and generate the system initial values based on the image hash value. Taking the Cameraman image as an example, its hash value *H* is 5bc100741511b04c58ef132109b9c6-d8c89d1355aa7b7eeabfb5bee1a9970d56. This H is then divided into 8-bit blocks $h_{i}$ in decimal format, which are expressed as follows:

(3) $$H=\{h_{1},h_{2},\ldots ,h_{32}\}$$

The initial dynamic key is constructed using $h_{1}$ to $h_{24}$, as shown in the following equation:

(4) $$\left\{\begin{array}{c} x_{0}=mod\left(\frac{h_{1}h_{2}h_{3}h_{4}h_{5}h_{6}}{10^{5}},1\right)\\ y_{0}=mod\left(\frac{{h_{4}h}_{5}h_{6}h_{7}h_{8}h_{9}}{10^{5}},1\right)\\ z_{0}=mod\left(\frac{{h_{7}h_{8}h}_{9}h_{10}h_{11}h_{12}}{10^{5}},1\right)\\ w_{0}=mod\left(\frac{{h_{10}h_{11}h_{12}h}_{13}h_{14}h_{15}}{10^{5}},1\right)\\ v_{0}=mod\left(\frac{{h_{13}h_{14}h_{15}h_{16}h}_{17}h_{18}}{10^{5}},1\right)\\ u_{0}=mod\left(\frac{{{h_{16}h}_{17}h_{18}h_{19}h_{20}h}_{21}}{10^{5}},1\right) \end{array}\right.$$

where $x_{0}$,$\;y_{0}$,$\;z_{0}$,$\;w_{0}$,$\;v_{0}$, and $u_{0}$ denote the initial values. Next, this algorithm generates four random numbers $r_{1}$ to $r_{4}$ within the interval [0, 255] using $h_{25}$ to $h_{32}$, as shown in the following equation:

(5) $$\left\{\begin{array}{c} r_{1}=mod\left(floor(h_{25}+h_{26}),256\right)\\ r_{2}=mod\left(floor(h_{27}+h_{28}),256\right)\\ r_{3}=mod\left(floor(h_{29}+h_{30}),256\right)\\ r_{4}=mod\left(floor(h_{31}+h_{32}),256\right) \end{array}\right.$$

Step 3: Set the system parameters as a=6, b=2, c=0.1, d=2, m=−1, n=−0.2, p=1 and q=0.1. The system (1) performs $sum\left(r\right)+M\times N$ iterations. When generating the pseudorandom sequences, the algorithm discards the first sum(r) iterations results to eliminate transient effects, thereby yielding pseudorandom sequences {$x_{i}$} and {$y_{i}$} of length M×N. Matrices G and H are then derived from these sequences using Equation (6).

(6) $$\left\{\begin{array}{c} G\left(i,j\right)=mod(floor((f_{1}\times x((i-1)\times N+j))\times 10^{13}),256)\\ H\left(i,j\right)=mod(floor((f_{2}\times y((i-1)\times N+j))\times 10^{13}),256) \end{array}\right.$$

where i=1,2,…,M; j=1,2,…,N; and $f_{1}$ and $f_{2}$ are given by Equation (7):

(7) $$\left\{\begin{array}{c} f_{1}=\frac{r_{1}}{r_{2}+r_{3}+1}\\ f_{2}=\frac{r_{2}}{r_{1}+r_{3}+1} \end{array}\right.$$

Step 4: The plaintext image P undergoes the first diffusion with matrices G and H using Equation (8), yielding the image matrix B:

(8) $$\left\{\begin{array}{c} B\left(1,1\right)=P\left(1,1\right){⊕T}_{3}\left(1,1\right)\\ B\left(i,j\right)=P\left(i,j\right){⊕T}_{3}\left(i,j\right) \end{array}\right.$$

where the expressions for $T_{3}(1,1)$ and $T_{3}(i,j)$ are given as follows:

(9) $$\left\{\begin{array}{c} T_{1}\left(1,1\right)=mod\left(r_{1}+G\left(1,1\right),256\right)\\ T_{2}\left(1,1\right)=mod\left(r_{2}+H\left(1,1\right),256\right)\\ T_{3}\left(1,1\right)=T_{1}\left(1,1\right)⊕T_{2}\left(1,1\right)\\ T_{1}\left(i,j\right)=mod\left(r_{1}+r_{2}+G\left(i,j\right),256\right)\\ T_{2}\left(i,j\right)=mod\left(r_{1}+r_{2}+H\left(i,j\right),256\right)\\ T_{3}\left(i,j\right)=T_{1}\left(i,j\right){⊕T}_{2}\left(i,j\right) \end{array}\right.$$

Step 5: The pixels of matrix B are rearranged via the Zigzag algorithm introduced in Section 2.2, yielding the initially scrambled image $A_{1}$.

Step 6: The second-level dynamic key $x_{0}^{\prime }$ and $z_{0}^{\prime }$ are computed using Equation (10):

(10) $$\left\{\begin{array}{c} k^{\prime }=mod(\left(1000\times (a+b+c+d)\right),256)\\ {x^{\prime }}_{0}=mod((x_{0}+\frac{{h_{22}h}_{23}}{256}+\frac{k^{\prime }}{256}),1)\\ {z^{\prime }}_{0}=mod({(z}_{0}+\frac{{h_{23}h}_{24}}{256}+\frac{k^{\prime }}{256}),1) \end{array}\right.$$

Similarly, the system iterates to eliminate transient effects and yields pseudorandom sequences {$z_{i}$} and {$w_{i}$} of length M×N. Matrices K and L are then derived from these sequences using Equation (11).

(11) $$\left\{\begin{array}{c} K(i,j)=mod(floor((f_{3}\times z((i-1)\times N+j))\times 10^{13}),256)\\ L(i,j)=mod(floor((f_{4}\times w((i-1)\times N+j))\times 10^{13}),256) \end{array}\right.$$

where i=1,2,…,M; j=1,2,…,N; and $f_{3}$ and $f_{4}$ are given by Equation (12):

(12) $$\left\{\begin{array}{c} f_{3}=\frac{r_{3}}{r_{1}+r_{4}+1}\\ f_{4}=\frac{r_{2}}{r_{3}+r_{4}+1} \end{array}\right.$$

Step 7: The image matrix $A_{1}$ undergoes the second diffusion with matrices K and L using Equation (13), yielding the image C:

(13) $$\left\{\begin{array}{c} C\left(M,N\right)=A_{1}\left(M,N\right){⊕T}_{6}\left(M,N\right)\\ C\left(i,j\right)=A_{1}\left(i,j\right){⊕T}_{6}\left(i,j\right) \end{array}\right.$$

where the expressions for $T_{6}(M,N)$ and $T_{6}(i,j)$ are given as follows:

(14) $$\left\{\begin{array}{c} T_{4}\left(M,N\right)=mod\left(r_{3}+K\left(M,N\right),256\right)\\ T_{5}\left(M,N\right)=mod\left(r_{4}+L\left(M,N\right),256\right)\\ T_{6}\left(M,N\right)=T_{4}\left(M,N\right){⊕T}_{5}\left(M,N\right)\\ T_{4}\left(i,j\right)=mod\left(r_{3}+r_{4}+K\left(i,j\right),256\right)\\ T_{5}\left(i,j\right)=mod\left(r_{3}+r_{4}+L\left(i,j\right),256\right)\\ T_{6}\left(i,j\right)=T_{4}\left(i,j\right){⊕T}_{5}\left(i,j\right) \end{array}\right.$$

Step 8: Similar to Step 6, the third-level dynamic key $x_{0}^{\prime \prime }$ is computed using Equation (15), which yields pseudorandom sequences {$v_{i}$} and {$u_{i}$} of length M×N.

(15) $$\left\{\begin{array}{c} k^{\prime \prime }=mod(\left(1000\times (m+n+p+q)\right),256)\\ {x^{\prime \prime }}_{0}=mod({{(x}^{\prime }}_{0}+\frac{{h_{22}h}_{24}}{256}+\frac{k^{\prime \prime }}{256}),1) \end{array}\right.$$

Step 9: The pixels of matrix C are rearranged via the chaotic index scrambling algorithm introduced in Section 2.3, with the sequence vector given by v+u, yielding the encrypted image D.

All encryption operations in the proposed encryption algorithm are reversible; consequently, the decryption algorithm corresponds to the inverse process of the encryption algorithm.

### 3.2. Experimental Results of Encryption Algorithm

**Figure 5:**
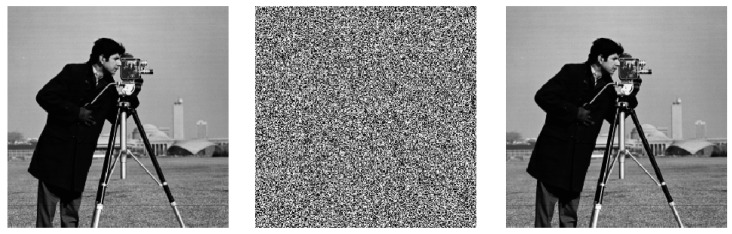

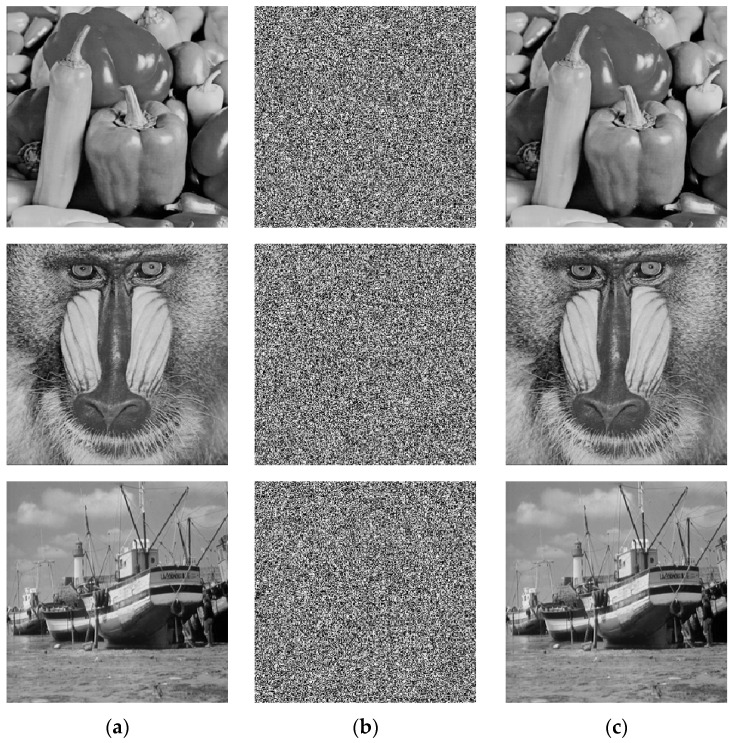
Encryption effect. (**a**) Original images; (**b**) Encrypted images; (**c**) Decrypted images.

The test images include two sets of standard grayscale images: one set with a resolution of 256 × 256 pixels, including Cameraman, Peppers, Baboon, and Boat; and the other set with a resolution of 512 × 512 pixels, featuring the same image content. The experimental environment is a Windows 11 operating system with an Intel^®^ Core (TM) i5-9300H CPU (2.40 GHz), 8 GB of RAM, and MATLAB R2020a. The encrypted and decrypted images from the simulations are presented in Figure 5, where column (a) shows original images, column (b) encrypted images, and column (c) decrypted images. The results show that the proposed algorithm effectively conceals the information of the plaintext image after encryption, and the decrypted image is identical to the plaintext image, thereby demonstrating the effectiveness and feasibility of the designed encryption algorithm.

## 4. Security Analysis

### 4.1. Key Space Analysis

The key space denotes the set of all potential keys for an encryption algorithm. Generally, an encryption algorithm can resist the brute-force attacks when its key space exceeds 2^100^ [24]. The key of the proposed algorithm comprises system parameters (a,b,c,d,m,n,p,q), initial dynamic key ($x_{0}$,$y_{0}$,$z_{0}$,$w_{0}$,$v_{0}$,$u_{0}$), and secondary and tertiary dynamic keys ($x_{0}^{\prime }$, $z_{0}^{\prime }$, $x_{0}^{\prime \prime }$). Since the precision of c, n, and q is 1 × 10^−16^, while that of a,b,d,m, and p is 1 × 10^−15^, the key space size of the proposed algorithm exceeds $10^{16\times 3+15\times 5}=10^{123}$. This size is far greater than the theoretical threshold of 2^100^, confirming that the proposed algorithm can effectively withstand exhaustive attacks.

### 4.2. Key Sensitivity Analysis

**Figure 6:**
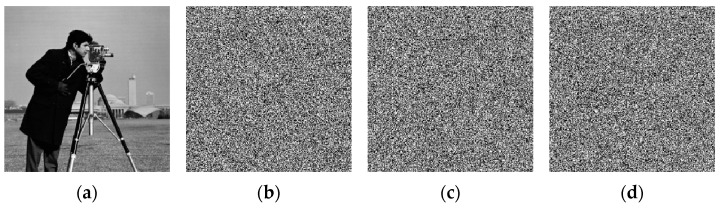
Key sensitivity test. (**a**) Original key; (**b**) a=a + × 10^−15^; (**c**) d=d + × 10^−15^; (**d**) m=m + × 10^−16^.

Key sensitivity is one of the critical factors for evaluating the security of image encryption algorithms. First, we encrypted the Cameraman image using the original key, yielding a fixed encrypted image. Next, taking a, d, and m as examples, we introduced slight modifications to the key: a=a+ 1 × 10^−15^, d=d+ 1 × 10^−15^, and m=m+ 1 × 10^−16^. Finally, while keeping the other input keys unchanged, we input each fine-tuned key, respectively. The resulting decrypted images are shown in Figure 6. The results demonstrate that images decrypted with the modified keys fail to recover the plaintext information, which confirms that the proposed algorithm exhibits strong key sensitivity.

### 4.3. Histogram Analysis

**Figure 7:**
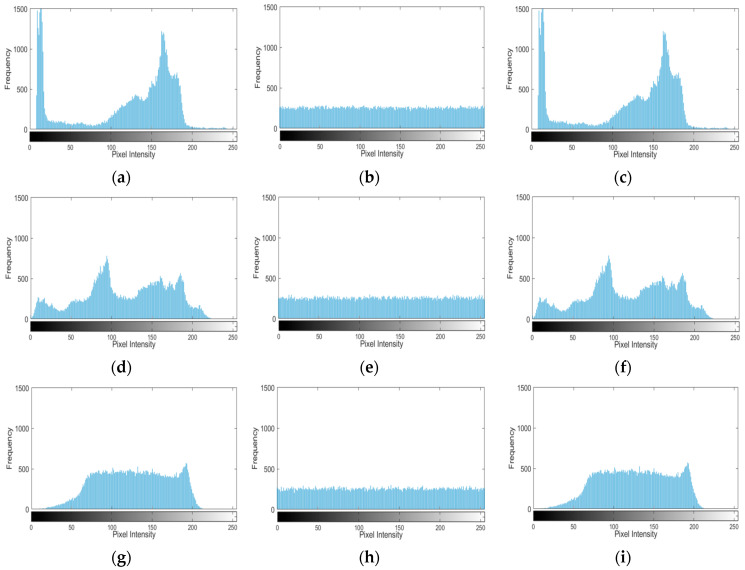

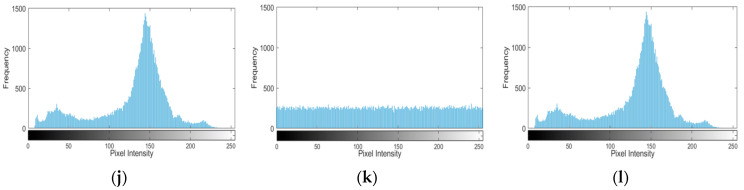
Histograms. (**a**–**c**) correspond to the original, encrypted, and decrypted histograms of Cameraman; (**d**–**f**) correspond to the original, encrypted, and decrypted histograms of Peppers; (**g**–**i**) correspond to the original, encrypted, and decrypted histograms of Baboon; (**j**–**l**) correspond to the original, encrypted, and decrypted histograms of Boat.

Histogram analysis is a critical method for assessing the resistance of an encryption algorithm to statistical analysis. Generally, the more uniform the pixel histogram of an image, the stronger its ability to withstand statistical analysis attacks. We perform quantitative analysis on the histogram using Equation (16):

(16) $$x^{2}=\sum_{i=0}^{255}\frac{{\left(O_{i}-\frac{N}{256}\right)}^{2}}{\frac{N}{256}}$$

where N denotes the total number of pixels in the image, $O_{i}$ represents the count of pixels with a pixel value of i, and i ϵ [0, 255]. Figure 7 presents the histograms of the original, decrypted, and encrypted images. The experimental results demonstrate that the ciphertext image exhibits a uniform pixel distribution, which differs significantly from that of the original image.

As shown in Table 1, the proposed encryption algorithm substantially reduces the $X^{2}$ statistic of the encrypted image. For each of the eight test images, the algorithm decreases the $X^{2}$ statistic of their corresponding ciphertext images by over 99.16% compared to the original images. These results confirm that the proposed algorithm can effectively resist statistical attacks.

### 4.4. Correlation Analysis

Pixel correlation describes the similarity between adjacent pixels. Generally, in plaintext images, adjacent pixels show high correlation in the vertical, horizontal, and diagonal directions. The equation [25] for calculating the correlation coefficient is as follows:

(17) $$\left\{\begin{array}{c} r^{\prime }=\frac{cov\left(x,y\right)}{\sqrt{D\left(x\right)}\sqrt{D\left(y\right)}}\\ cov\left(x,y\right)=\frac{1}{n}\sum_{i=1}^{n}\left(x_{i}-E_{x}\right)\left(y_{i}-E_{y}\right)\\ D\left(x\right)=\frac{1}{n}\sum_{i=1}^{n}\left(x_{i}-E_{x}\right)\left(y_{i}-E_{y}\right)\\ E\left(x\right)=\frac{1}{n}\sum_{i=1}^{n}x_{i} \end{array}\right.$$

where cov(x,y) is the covariance of x and y; r′ is the correlation coefficient; D(x) and D(y) are the variances of x and y, respectively; $x_{i}$ and $y_{i}$ are the observed values of x and y at pixel i; $E_{x}$ and $E_{y}$ are the mathematical expectations of x and y, respectively; and n is the number of pixels.

**Figure 8:**
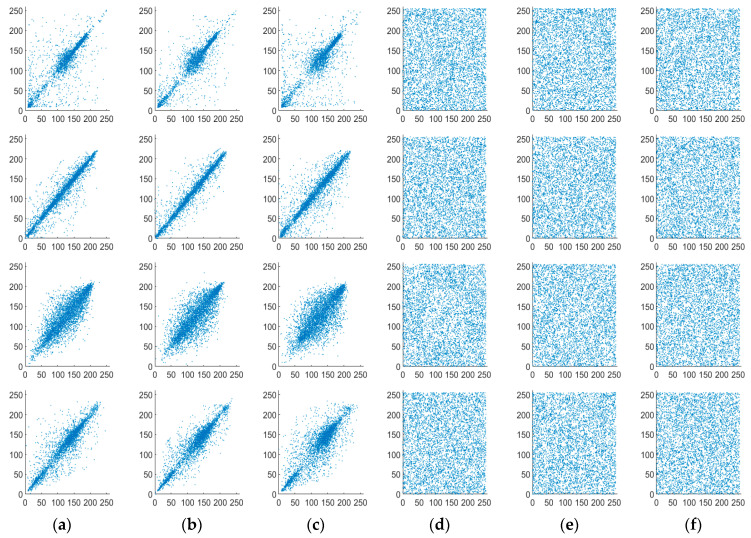
Image adjacent pixel distribution map. (**a**) Plaintext horizontal direction; (**b**) Plaintext vertical direction; (**c**) Plaintext diagonal direction; (**d**) Ciphertext horizontal direction; (**e**) Ciphertext vertical direction; (**f**) Ciphertext diagonal direction.

Correlation comprises positive and negative correlation. If the adjacent pixels are strongly correlated, the correlation coefficient will be close to ±1. Conversely, the coefficient approaching 0 indicates no correlation. Using Cameraman, Peppers, Baboon, and Boat as test images, we randomly selected 5000 pixel pairs from the original and encrypted versions of each image, performed correlation tests, and calculated the correlation coefficients for all directions. In Figure 8, columns (a), (b), and (c) correspond to the adjacent pixel correlation plots of the plaintext images in each direction, while columns (d), (e), and (f) correspond to those of the ciphertext images. As shown in the figure, the pixel distribution in the ciphertext image is more uniform, which means the distribution of adjacent pixels is more random.

Table 2 lists the correlation coefficients of the test images in the horizontal, vertical, and diagonal directions, while Table 3 shows a comparison of the correlation coefficients between the proposed algorithm and those from other literature. The results demonstrate that the average of the absolute values of the correlation coefficients is smaller than the corresponding values of other algorithms, indicating that the algorithm effectively breaks the adjacent pixel correlation in plaintext images.

### 4.5. Information Entropy Analysis

In image encryption, information entropy is a critical metric for assessing the complexity and randomness of an image [28]. The closer the information entropy of a ciphertext is to the ideal value of 8, the higher the uncertainty of the image is. The information entropy values of the image before and after encryption were calculated separately using Equation (18).

(18) $$H\left(G\right)=\sum_{i=1}^{L}P(G_{i}){log}_{2}\frac{1}{P(G_{i})}$$

where $G_{i}$ is the grayscale value of image pixels, $P(G_{i})$ denotes the probability of $G_{i}$ occurring in the image, and L is the grayscale level. Table 4 shows the information entropy results of the proposed algorithm, while Table 5 presents the comparison results of the experimental image entropy with those from other literature. The results demonstrate that the information entropy of images encrypted by the proposed algorithm is closer to the ideal value, meaning the encrypted images are more resistant to attacks.

### 4.6. Differential Attack

The ability to resist differential attacks is typically a critical metric for evaluating the plaintext sensitivity of an algorithm. Plaintext sensitivity means that when the pixel at a certain position in a plaintext image changes, the corresponding ciphertext image should undergo significant changes compared to the original ciphertext image. This change is measured using the Number of Pixels Change Rate (NPCR) and the Unified Average Changing Intensity (UACI). The equation for calculating NPCR and UACI are as follows:

(19) $$NPCR=\frac{\sum_{i=1}^{M}\sum_{j=1}^{N}D(i,j)}{M\times N}\times 100\%$$

(20) $$UACI=\frac{\sum_{i=1}^{M}\sum_{j=1}^{N}\left|C_{1}\left(i,j\right)-C_{2}\left(i,j\right)\right|}{M\times N\times 255}\times 100\%$$

(21) $$D\left(i,j\right)=\left\{\begin{array}{c} 0,C_{1}\left(i,j\right)=C_{2}\left(i,j\right)\\ 1,C_{1}\left(i,j\right)\neq C_{2}\left(i,j\right) \end{array}\right.$$

Where M and N denote the row and column counts of an original image, respectively. $C_{1}\left(i,j\right)$ and $C_{1}\left(i,j\right)$ represent the encrypted images corresponding to a plaintext image before and after a random pixel value change, respectively. For 256-level grayscale images, the ideal values of NPCR and UACI are 99.6094% and 33.4635% [33]. In each test, we randomly selected one or multiple pixel positions, incremented their pixel values by 1, repeated the experiment 100 times, and then calculated the average. Table 6 presents the differential attack resistance analysis results of the proposed algorithm when one pixel and five pixels are fine-tuned, while Table 7 shows the comparison results of the Cameraman image’s performance with those from other literature. The results indicate that the measured NPCR and UACI of the proposed algorithm are closer to the ideal values, demonstrating that the algorithm is highly sensitive to plaintext images and can effectively resist differential attacks.

### 4.7. Anti-Noise Capability Analysis

**Figure 9:**
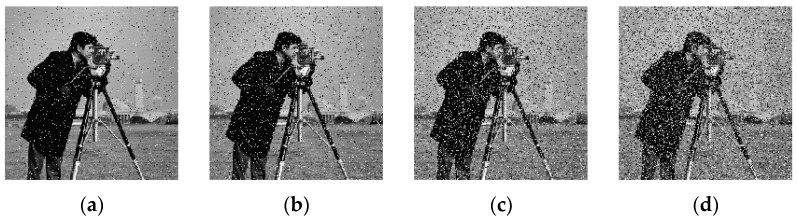
Salt-and-pepper noise attack with (**a**) intensity 0.05; (**b**) 0.1; (**c**) 0.2; (**d**) 0.3.

During image transmission, images may be contaminated by noise, which may render them undecryptable. To ensure the robustness of an image encryption algorithm, it should possess the capability to resist noise attacks [36]. To test the noise resistance of the algorithm, we added salt-and-pepper noise with intensities of 0.05, 0.1, 0.2, and 0.3 to the experimental ciphertext image, respectively, then performed decryption for each case. Figure 9 shows the decrypted results after introducing salt-and-pepper noise of different proportions to the ciphertext. From the simulations, even after noise attacks on the ciphertext, key information remains relatively clear in the decrypted image. This demonstrates that the proposed algorithm exhibits strong robustness, thereby ensuring images can tolerate a certain level of noise contamination during transmission.

To better evaluate and compare the noise attack resistance of the algorithm, we conducted a quantitative analysis by calculating the peak signal-to-noise ratio (PSNR), whose calculation formula is as follows:

(22) $$PSNR=10\times \log_{10}\left(\frac{255^{2}}{MSE}\right)$$

(23) $$MES=\frac{1}{mn}\sum_{i=1}^{m}\sum_{j=1}^{n}{\left(P\left(i,j\right)-D(i,j)\right)}^{2}$$

where m and n stand for the width and height of the image, respectively. P(i,j) denotes the pixel value at position (i, j) in the plain image and D(i,j) represents the pixel value at the same position (i,j) in the decrypted image. A higher PSNR indicates less distortion between the original image and the decrypted image, demonstrating that the algorithm exhibits stronger robustness against noise. Table 8 shows the experimental results, which indicate that the proposed algorithm exhibits superior PSNR performance compared with other algorithms, demonstrating that the algorithm achieves effectiveness in resisting noise attacks.

### 4.8. Cropping Attack Analysis

**Figure 10:**
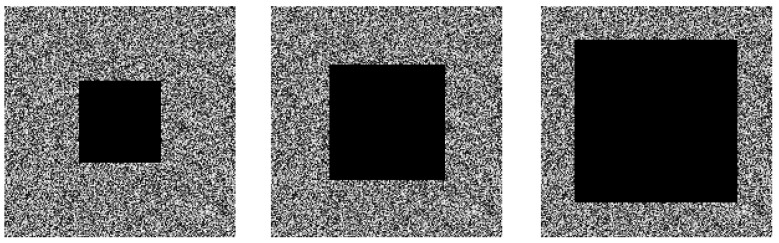

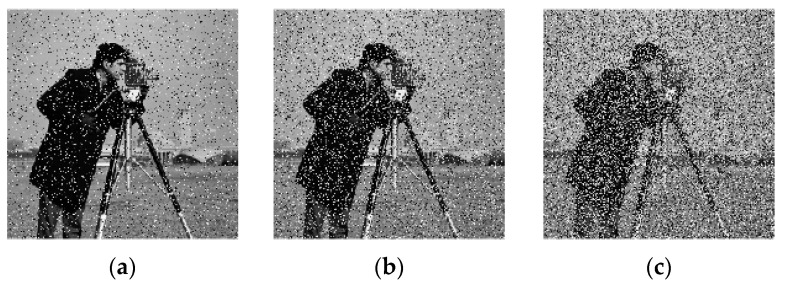
Cropping attacks on ciphertext. (**a**) Cut 1/8; (**b**) Cut 1/4; (**c**) Cut 1/2.

During image transmission, data loss may occasionally occur, preventing the retrieval of accurate original image information after decryption. To ensure the robustness of an image encryption algorithm, it must resist cropping attacks. To test the ability of the proposed algorithm to withstand data loss, we cropped the Cameraman ciphertext image at ratios of 1/8, 1/4, and 1/2, respectively, then performed decryption. Figure 10 shows the decrypted images after cropping the ciphertext at these different ratios. From the simulation results, when the ciphertext suffers 1/8 or 1/4 data loss, key image information remains clearly visible in the decrypted result; with 1/2 data loss, while most original image information is unrecoverable post-decryption, the general outline of the image is still distinguishable. Furthermore, Table 9 shows the PSNR values between the decrypted images and the original images after cropping the ciphertexts of the experimental images at different ratios; the results indicate that the proposed algorithm exhibits excellent PSNR performance. This demonstrates that the proposed algorithm exhibits strong robustness, enabling it to tolerate a certain amount of data loss during image transmission.

### 4.9. Algorithm Efficiency Analysis

In practical applications, image encryption algorithms demand high efficiency. Table 10 shows the encryption times of all experimental images. Additionally, we compared the encryption time of the images with results from other literature. It can be observed that our proposed algorithm has a shorter encryption time than those in the literature [35,40,41]. This indicates the proposed algorithm not only enhances encryption security but also maintains favorable encryption efficiency.

### 4.10. Chosen-Plaintext Attack Analysis

The ability to resist chosen-plaintext attacks is an important indicator for measuring an image encryption algorithm. We use the Formula (24) to verify whether the proposed algorithm is resistant to chosen-plaintext attacks [42]. If the obtained image results demonstrate that this formula does not hold, it indicates that the algorithm has a good ability to resist chosen-plaintext attacks.

(24) $$P_{1}\left(i,j\right)⊕{\;P}_{2}\left(i,j\right)=C_{1}\left(i,j\right)⊕{\;C}_{2}\left(i,j\right)$$

where $P_{1}(i,j)$ and $P_{2}(i,j)$ represent the plaintext images, and $C_{1}(i,j)$ and $C_{2}(i,j)$ are the ciphertext images.

**Figure 11:**
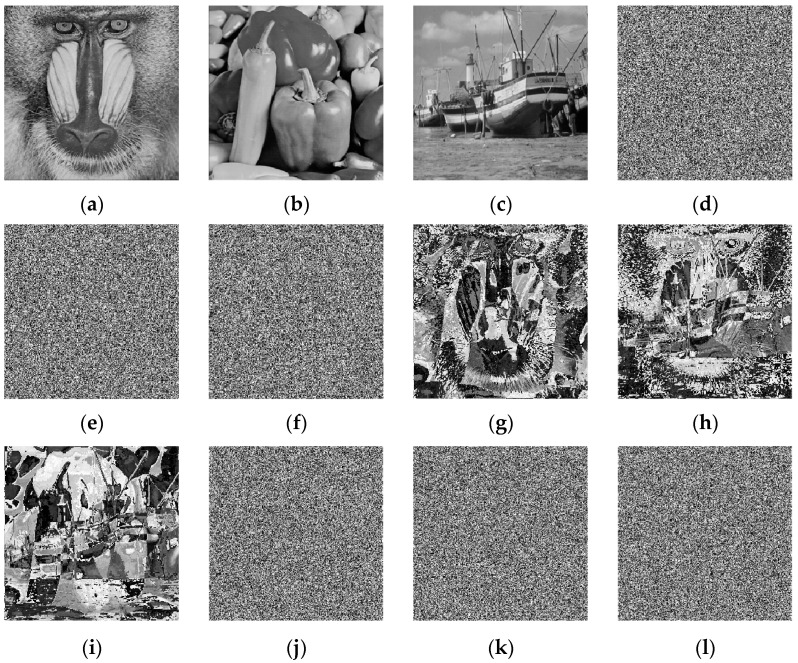
Chosen-plaintext attack. (**a**) Baboon; (**b**) Peppers; (**c**) Boat; (**d**) Encrypted Baboon; (**e**) Encrypted Peppers; (**f**) Encrypted Boat; (**g**) Baboon xor Peppers; (**h**) Baboon xor Boat; (**i**) Peppers xor Boat; (**j**) Encrypted Baboon xor encrypted Peppers; (**k**) Encrypted Baboon xor encrypted Boat; (**l**) Encrypted Peppers xor encrypted Boat.

We used 256 × 256 grayscale images Baboon, Peppers, and Boat. As shown in Figure 11, the XOR results between any two plaintext images are inconsistent with the corresponding results of ciphertext images, indicating that Equation (24) does not hold. In addition, a 256-bit hash value is generated after the image is processed by the hash function. Even if there is only one bit difference in the original image, the generated hash value will be completely different. Meanwhile, the initial values of the chaotic system are determined by the plaintext, and the algorithm applies the pseudorandom sequences generated by the system in the encryption process, making the algorithm highly sensitive to the original image. Thus, small changes in the original pixel values will trigger an avalanche effect, and there are no equivalent keys in the proposed algorithm.

Table 11 shows that after the XOR operation between any two ciphertext images, the information entropy of the resulting image remains close to the ideal value, and its pixel correlation coefficients approach 0. This indicates that the image exhibits high randomness, and attackers cannot obtain any identifiable information from the resulting image. Thus, the proposed algorithm can effectively resist chosen-plaintext attacks.

## 5. Conclusions

This study proposes an image encryption algorithm based on a six-dimensional memristive chaotic system, Zigzag scrambling, and improved chaotic index scrambling. We use plaintext-independent system parameters as input keys and associate the generation of sequences with the plaintext. Meanwhile, we adopt a hierarchical key method to enhance the security of the algorithm. This scheme has application value in scenarios such as image transmission and privacy protection. Experimental analysis results show that compared with existing studies, the proposed algorithm exhibits excellent performance in key space, key sensitivity, information entropy, and encryption efficiency. Additionally, it can resist common attacks, including statistical, differential, noise, cropping, and chosen-plaintext attacks. Given the diverse requirements across different scenarios in cryptography for practical applications, future work will investigate the performance of the algorithm on color images. Additionally, while ensuring the security of the algorithm, we will optimize encryption efficiency to achieve further improvements.

## Figures and Tables

**Table 1:** $X^{2}$ statistic results.

Images	Image Size	Plain	Cipher	Reduction
Cameraman	256 × 256	110,973.30	186.6875	99.8318%
Peppers		31,988.95	268.0859	99.1619%
Baboon		42,256.09	255.5156	99.3953%
Boat		100,313.13	263.1250	99.7380%
Cameraman	512 × 512	420,990.80	235.6230	99.9440%
Peppers		127,496.13	242.3477	99.8099%
Baboon		175,925.14	277.9355	99.8420%
Boat		406,895.03	263.2461	99.9353%

**Table 2:** Correlation coefficient.

Images	Direction	Plaintext	Ciphertext	Reduction
Cameraman (256 × 256)	Horizontal	0.9335	−0.0044	99.5287%
	Vertical	0.9592	−0.0031	99.6768%
	Diagonal	0.9087	0.0060	99.3397%
Peppers (256 × 256)	Horizontal	0.9620	−0.0036	99.6258%
	Vertical	0.9698	0.0048	99.5051%
	Diagonal	0.9357	−0.0033	99.6473%
Baboon (256 × 256)	Horizontal	0.8529	0.0045	99.4724%
	Vertical	0.8163	−0.0001	99.9877%
	Diagonal	0.7709	0.00001	99.9987%
Boat (256 × 256)	Horizontal	0.9171	0.0020	99.7819%
	Vertical	0.9391	0.0036	99.6167%
	Diagonal	0.8708	−0.0011	99.8737%
Cameraman (512 × 512)	Horizontal	0.9834	−0.0002	99.9797%
	Vertical	0.9903	−0.0020	99.7980%
	Diagonal	0.9737	0.0011	99.8870%
Baboon (512 × 512)	Horizontal	0.9647	−0.0003	99.9689%
	Vertical	0.9546	0.0004	99.9581%
	Diagonal	0.9273	−0.0035	99.6226%

**Table 3:** Comparison of images correlation coefficient with other algorithms.

Images	Algorithm	Plain			Cipher			
		Horizontal	Vertical	Diagonal	Horizontal	Vertical	Diagonal	Average
Cameraman (512 × 512)	Ours	0.9834	0.9903	0.9737	−0.0002	−0.0020	0.0011	0.0011
	[26]	0.9834	0.9902	0.9737	−0.0034	0.0009	−0.0014	0.0019
	[27]	0.9829	0.9898	0.9730	−0.0038	−0.0051	0.0004	0.0031
Peppers (512 × 512)	Ours	0.9904	0.9924	0.9823	−0.0019	0.0013	0.0019	0.0017
	[26]	0.9768	0.9792	0.9636	−0.0004	0.0031	−0.0031	0.0022
	[27]	0.9805	0.9829	0.9655	−0.0046	−0.0052	0.0001	0.0033
Baboon (512 × 512)	Ours	0.9647	0.9546	0.9273	−0.0003	0.0004	−0.0035	0.0014
	[26]	0.8665	0.7587	0.7262	0.0001	−0.0001	0.0045	0.0016
	[27]	0.9317	0.9105	0.8650	−0.0067	−0.0058	0.0027	0.0051

**Table 4:** Information entropy.

Images	Image Size	Plaintext	Ciphertext
Cameraman	256 × 256	7.0097	7.9979
Peppers		7.5797	7.9971
Baboon		7.3715	7.9972
Cameraman	512 × 512	7.0482	7.9994
Peppers		7.5808	7.9993
Baboon		7.3468	7.9992

**Table 5:** Comparison of information entropy with other algorithms.

Images	Image Size	Ref. [26]	Ref. [27]	Ref. [29]	Ref. [30]	Ref. [31]	Ref. [32]	Proposed
Cameraman	256 × 256	-	-	7.9972	7.9968	7.9973	7.9976	7.9979
Peppers		-	-	7.9971	7.9971	7.9972	-	7.9971
Baboon		-	-	-	7.9970	7.9970	-	7.9972
Cameraman	512 × 512	7.9993	7.9988	-	-	7.9993	7.9993	7.9994

**Table 6:** NPCR and UACI.

Size	Parameter	Cameraman	Peppers	Baboon	Boat	Average
256 × 256	NPCR (%)	99.6111	99.6083	99.6091	99.6104	99.6097
	UACI (%)	33.4643	33.4580	33.4463	33.4840	33.4631
512 × 512	NPCR (%)	99.6099	99.6114	99.6085	99.6091	99.6097
	UACI (%)	33.4554	33.4601	33.4949	33.4354	33.4615
256 × 256	NPCR (%)	99.6102	99.6152	99.6093	99.6134	99.6120
	UACI (%)	33.4602	33.4616	33.4344	33.4780	33.4586
512 × 512	NPCR (%)	99.6071	99.6096	99.6093	99.6117	99.6094
	UACI (%)	33.4637	33.4880	33.4827	33.4239	33.4646

**Table 7:** Comparison of the Cameraman’s performance with other algorithms.

Parameter	Proposed	Ref. [29]	Ref. [34]	Ref. [35]	Ideal Value
NPCR (%)	99.6111	99.6078	99.64	99.6017	99.6094
UACI (%)	33.4643	33.5237	33.39	33.3855	33.4635

**Table 8:** Robustness analysis against noise attack.

Algorithm	Images	Size	Noise Intensity	PSNR (dB)
Ours	Cameraman	256 × 256	0.05	21.3757
			0.1	18.4846
			0.2	15.3956
			0.3	13.6608
	Baboon	256 × 256	0.05	22.5374
			0.1	19.3729
			0.2	16.3605
			0.3	14.6581
	Boat	256 × 256	0.05	22.4760
			0.1	19.2591
			0.2	16.3732
			0.3	14.6581
	Cameraman	512 × 512	0.05	21.3916
			0.1	18.3453
			0.2	15.3829
			0.3	13.6757
	Baboon	512 × 512	0.05	22.4504
			0.1	19.5186
			0.2	16.4893
			0.3	14.7262
[29]	Cameraman	256 × 256	0.05	17.8969
			0.1	15.0925
			0.2	12.5765
			0.3	11.1662
[31]	Cameraman	512 × 512	0.005	37.64
[37]	Cameraman	512 × 512	0.1	18.2589
	Baboon		0.1	19.2231
[38]	Cameraman	256 × 256	0.05	18.58
	Peppers	256 × 256	0.05	19.14
	Boat	512 × 512	0.05	19.46
[39]	Boat	256 × 256	0.05	22.3642
			0.1	19.1878

**Table 9:** Robustness analysis against cropping attack.

Algorithm	Images	Size	Ratio	PSNR
Ours	Cameraman	256 × 256	1/8	17.5778
			1/4	14.4552
			1/2	11.4224
	Peppers	256 × 256	1/8	17.9909
			1/4	14.9162
			1/2	11.9250
	Cameraman	512 × 512	1/8	17.3866
			1/4	14.4083
			1/2	11.4200
	Peppers	512 × 512	1/8	17.9319
			1/4	14.9453
			1/2	11.9323
	Boat	512 × 512	1/8	18.3399
			1/4	15.3719
			1/2	12.3661
[38]	Cameraman	256 × 256	1/2	11.41
	Peppers	256 × 256	1/2	11.89
	Boat	512 × 512	1/2	12.31
[37]	Cameraman	512 × 512	1/4	14.6359
			1/2	11.5825
	Peppers	512 × 512	1/4	14.1524
			1/2	11.2952
[32]	Goldhill	512 × 512	1/4	14.6848
			1/2	11.7152

**Table 10:** Comparison of encryption time.

Images	Algorithm	Encrypted Time (s)
Cameraman	Ours (256 × 256)	0.342
Peppers		0.353
Baboon		0.341
Boat		0.336
Cameraman	Ours (512 × 512)	1.393
Peppers		1.413
Baboon		1.522
Boat		1.418
Cameraman	[41] (256 × 256)	0.41
	[35] (256 × 256)	0.421
	[40] (256 × 256)	0.3651
Baboon	[41] (256 × 256)	0.38

**Table 11:** Encrypted results of test images (⊕ denotes the XOR operation).

Test Images	Information Entropy	$X^{2}$	Correlation Coefficients		
			Horizontal	Vertical	Diagonal
Baboon ⊕ Peppers	7.9975	227.56	0.0035	−0.0050	−0.0056
Baboon ⊕ Boat	7.9972	255.87	0.0045	−0.0046	0.0028
Peppers ⊕ Boat	7.9973	248.27	0.0026	0.0121	0.0043

## Data Availability

The data presented in this study are available on request from the corresponding author. The data are not publicly available due to privacy restrictions.
